# Within and Between-Leg Oil Transfer in an Oil Bee

**DOI:** 10.1093/iob/obaf025

**Published:** 2025-06-11

**Authors:** J Homburger, M Pineirua, J Casas, T Speck, F Gallenmüller

**Affiliations:** Plant Biomechanics Group & Botanic Garden, University of Freiburg, Freiburg D-79104, Germany; Freiburg Materials Research Center (FMF), University of Freiburg, Freiburg D-79104, Germany; Institut de Recherche sur la Biologie de l'Insecte, UMR CNRS 7261, Université de Tours, Tours F-37200, France; Institut de Recherche sur la Biologie de l'Insecte, UMR CNRS 7261, Université de Tours, Tours F-37200, France; Cluster of Excellence *liv*MatS, University of Freiburg, Freiburg D-79110, Germany; Plant Biomechanics Group & Botanic Garden, University of Freiburg, Freiburg D-79104, Germany; Freiburg Materials Research Center (FMF), University of Freiburg, Freiburg D-79104, Germany; Cluster of Excellence *liv*MatS, University of Freiburg, Freiburg D-79110, Germany; Plant Biomechanics Group & Botanic Garden, University of Freiburg, Freiburg D-79104, Germany

## Abstract

Oil bees, which gather oil from flowers, transfer the floral oil collected from fore-legs, to middle-legs, and then to hind-legs, where they store the oil until release in the nest's brood cell. The complex leg maneuvers and the specialized hair types according to their location and function on the legs have been described in the past using morphological observations and *in vivo* behavioral monitoring, sometimes during flight. The aim of this work is to describe the different steps of oil transfer and to infer the role of the different hair types using a manipulative approach on isolated legs, controlled amounts of oil and high speed video recordings. A rapid and uni-directional capillary oil movement from the collecting ventral side of tarsi and/or tibiae to their dorsal side is observed in each fore-, middle-, and hind-legs. This suggests that plumose setae and pluridentate setae present different functionalities, acting either as oil donors or receptors, depending on their location on the legs. In the transfers observed, very little oil remains on the donor surface, so that a bee collecting oil from flower can quickly replenish the donor surface again.

## Introduction

Coevolution has driven a variety of relationships between zoogamous plant species and their pollinators. Throughout the entire developmental history of today's flora and fauna numerous “benefit and reward systems” have evolved, including nutrients offered by the plants and collected by the pollinators as food for themselves or for their offspring. Whereas, in the majority of zoogamous plant species, such nutrients consist of pollen grains or sugary exudates, in some species rewards containing fatty acids have evolved, yielding to considerably more energy by digestion than sugars [estimated by Buchmann (1987) as 37.6 vs. 16.7 J/g]. The South-African orchid *Satyrium longicauda* can even switch from nectar production to flower oil production, accompanied by a change of visiting pollinator species (Renner 2021). Currently known floral oil-producing plant species have evolved within the orders of *Calceolariaceae*, *Cucurbitaceae*, *Iridaceae*, *Krameriaceae*, *Malpighiaceae*, *Myrsinaceae*, *Scrophulariaceae*, *Solanaceae*, and *Stilbaceae* (Vogel 1974,^ 1986^; Neff and Simpson 1981; Renner and Schaefer 2010).

Independent of the taxonomic classification, 2 different types of oil-producing and oil-bearing flower organs are distinguishable, according to the flower morphology and oil gland position, the epithelial and trichome elaiophores (Vogel 1974). Epithelial elaiophores are oil glands arranged as epithelium that secrete oil in a subcuticular space with gland cells protected and covered by a cuticula (Vogel 1974). Trichome elaiophores are flower areas with more or less densely arranged glandular oil trichomes. Their heads consist of several cells sometimes coated by a cuticula and secreting oil in a subcuticular space (Vogel 1974). In the different pollinator species, collecting organs are adapted to oil collection of the different elaiophore types. Elaiophores presenting the oil uncovered are often visited by oil-bees possessing setae with a broad base and highly branched apical ends (“lamellar pluridentate setae”). Elaiophores covered with a cuticula are mostly collected by oil-bees possessing less complex setae structures (“spatulate setae”). While bees possessing lamellar pluridentate setae visited in over 90% of the occasions the same plant species, bees with less complex setae showed less selectivity in plant species (Kuhlmann and Hollens 2014).

**Fig. 1.**
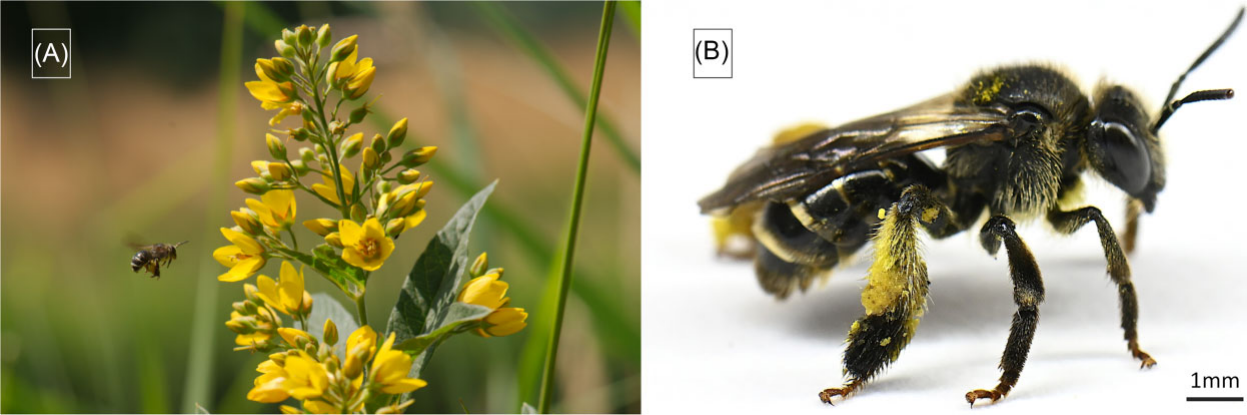
(**A**) *Macropis europaea* female visiting *L.*  *vulgaris*-flowers at a riverbank, (**B**) *M*. *europaea* female with storage structures on hind-legs loaded with pollen and oil.

In Europe, some species of *Lysimachia* (Primulaceae) produce floral oil in trichome elaiophores and are pollinated by oil-bees of the genus *Macropis* (Macropini; Mellitidae) (Fig. 1). These possess lamellar pluridentate setae on their legs (Vogel 1986) similar to those described by Kuhlmann and Hollens (2014) in South African *Rediviva* species.

Most oil-bees mix the collected floral oil with pollen and feed their larvae with the oil-pollen mixture. The pure oil furthermore serves as waterproof lining of the subterranean brood cells, isolating the brood cell and protecting the egg from moisture, fungi, and pathogens (Vogel 1986, Buchmann 1987, Vinson et al. 1996, Jesus and Garófalo 2000, Celary 2004, Martins et al. 2013). Oil-bees of both genera transfer the floral oil from their legs to the brood cell wall by sweeping movements, most likely with their distal tibia combs (Kuhlmann 2014). These hind-legs movements were also reported by Dötterl and Schäfer (2011). After the preparation of the brood cells the *Macropis* females start to collect pollen and oil in about 7 collecting flights on average. When the brood cell is filled with the pollen-oil-mixture they lay an egg inside and close the brood cell entrance (Schäffler and Dötterl 2010).

Female *Macropis* oil-bees are able to collect oil and pollen separately (Vogel 1986, Schäffler and Dötterl 2010). They primarily collect oil from the trichome elaiophores of *Lysimachia* oil-flowers using the ventral surfaces of their fore- and middle-legs and store this oil on the hind-leg scopae during foraging flights. The transfer from the fore- and middle-legs to the hind legs occurs while the bee hovers and was described by Vogel (1986) as backloading. It can be described as an iteration of specific movements. However, this backloading process is not yet understood in detail.

Vogel (1986) discussed a capillary gradient between the setae-pads of the different legs in combination with squeezing leg movements as mechanism underlying the oil transfer from leg to leg. Accordingly, upon contact between the legs oil flows from fore-leg to middle-leg and then hind-leg due to an increase in capillary force. Vogel (1986) also discussed a potentially dominant role of the notches and serrated comb-likes structures present between the tibia and trochanter in varying forms on the fore-, middle-, and hind-legs of female *M.*  *europaea* bees during the backloading process, but later rejected this hypothesis (Vogel 2002).

Our study is part of a larger project including an analysis of the physico-chemical properties of the setae covering the bee's legs and their function in different processes of oil transport and aiming at an implementation of these functional principles in technical biomimetic material systems. Here, we report on the oil-transporting structures in fore-, middle-, and hind-legs of female *M.*  *europaea* bees and observations of oil transports within single legs as well as between legs. The study builds upon the work of Vogel (1974, 1986, 2002) and continues it with a detailed analysis of all the single steps involved in the oil transport from the collecting organs in the front legs to the storage on the hind legs, which haven't been described and understood so far. Our approach distinguishes between unidirectional and passive oil transports occurring spontaneously and oil transports necessitating an energetic input by the bee, and correlate these fundamentally different processes to the functional morphology of the seta types involved. As the oil movements themselves cannot be observed from videos, even not from high-speed videos with a high resolution, due to their speed and the small dimensions of the setae, we studied the oil transport in different laboratory setups with individual legs.

## Material and methods

### Sample collection and preparation

*Macropis europaea* bees were caught in different locations around Freiburg im Breisgau, Germany (48.081832, 7.893776 and 48.009032, 7.858502). Prior to experimental manipulation the bee's legs were cleaned as follows: Fixation in 1:10 ethanol/H_2_O, 10 min ultrasound at 35°C, Transfer to 5:10 ethanol/H_2_O 20 min, 30 min ultrasound at 35°C, Transfer to 10:0 Ethanol/H_2_O 60 min, 60 min ultrasound at 35°C, Drying of the samples for 12 h at 60°C.

### Scanning electron microscopy

To analyze the morphology of the bee legs, a Scanning Electron Microscope (Zeiss Evo MA 15, Carl Zeiss AG, Oberkochen, Germany) was used on 5 nm gold coated (Cressington Sputter Coater 108auto, Tescan GmbH, Dortmund Germany) samples of *M. europaea* legs.

### Preparation of bee legs and video recordings of oil transfer

Fore-, middle-, and hind-legs of female *M.*  *europaea* bees were cut at the coxa and legs fixed with super glue (Everglue, Big Difference GmbH, Bad Bramstedt, Germany) onto flattened wires or toothpicks. The prepared fore- and middle-legs were loaded on the ventral surfaces with $0.15-0.2\mu l$ of sunflower oil (viscosity: $69\ {\mathrm{m}}{{\mathrm{m}}}^2{{\mathrm{s}}}^{ - 1}$ at $20^\circ {\mathrm{C}}$; density: $0.919{\mathrm{kg}}/{\mathrm{lL}}$; “Gut und Günstig,” Edeka Zentrale Stiftung & Co. KG, Hamburg, Germany) using a microsyringe. We chose to use sunflower oil because, according to Vogel (1986), most of its main chemical components are present in the floral oil in *Lysimachia vulgaris*, too. The loaded legs were attached to micromanipulators and manually moved until a contact between them was established and the subsequent oil transfer recorded with a Nikon Z8 (Nikon Corporation, Tokyo, Japan) and a custom macro lens setup constructed of a Nikon Telezoom 70–300 mm, 4.5–5.6f and a microscope lens Olympus 10x NA 0.25 (Olympus Corporation, Tokyo, Japan). In order to verify our observations in bees in their natural habitat, we put the legs into contact between (1) dorsal sides of fore-legs tarsi and ventral sides of tarsi and tibiae of middle-legs in these experiments, (2) ventral sides of middle-legs with the outside of the hind-legs, and (3) dorsal sides of the middle-legs with ventral sides of the hind-legs. As under natural conditions the transfer of oil among legs involves a series of leg movements of partially unknown nature and too complex for simulation, we induced the oil transfer by simply putting the legs into contact with each other in the described orientations under application of a light contact pressure in our experiments.

The backloading of oil from fore- to middle- to hind-legs in female *M.*  *europaea* bees were filmed in the field using a Nikon Z8 (Nikon Corporation, Tokyo, Japan) with a Nikon AF-S Nikkor Micro 105 mm/2.8 G IF-ED VR (Nikon Corporation, Tokyo, Japan) with 2160p and 120 fps. Cleaning movements were recorded in a caught bee with the same setup, but with lower resolution and frame rates (1080p, 60 fps).

## Results

### Morphology of bee legs

The setae pattern on legs of female *M.*  *europaea* bees differ significantly between the dorsal and ventral side, between tarsus and tibia and between different legs (Figs. 3 and 4). Here, we use the denominations adopted by Kuhlmann (2014) for the setae found in legs of *Rediviva* bees (lamellar pluridentate setae, spatula-shaped setae) and by Vogel (1986) for setae found in *Macropis* bees (plumose setae). Both tarsus (Fig. 3A) and tibia of fore-legs (Fig. 5) are covered with spatula-shaped setae (Fig. 4A) and lamellar pluridentate setae (Fig. 4B) on the ventral sides. Additionally, highly branched lamellar pluridentate setae are present on ventral sides of all tarsal segments and the pretarsus. They are arranged in a ridge-like pattern and surrounded by the spatula-shaped setae (Fig. 4A). In the fore-legs, the oil-collecting area comprises the ventral sides of the tarsus segments. Here, the spatula shaped (Figs. 3A and 4A: “sp”) and the lamellar pluridentate setae (Figs. 3A and 4B: “plr”) are forming a concave surface with their tips (Fig. 5A), the outer setae being longer than the central setae. The spatula-shaped setae in the oil-collecting area on the ventral sides of the tarsomeres are denser and shorter than those on the ventral side of the tibiae (Fig. 5A). Dorsal sides of both tarsal segments and tibia are covered by plumose setae (Fig. 3A: “plm,” Fig. 4E) interspersed by conical and apparently rigid setae (Fig. 4C). Tibiae in fore-legs have an oval cross-section, as described by Vogel (1986).

In the transition zones between ventral and dorsal sides, all types of setae are present, comparable to the intergradation zones described by Simpson et al. (1990) in *Centris* bees. This holds for the tarsal segments in fore-legs as well as for both tarsus and tibia in middle-legs (Figs. 3B and 6). Within the transition between tibia and femur a large notch is visible in fore-legs (Fig. 5B) and middle-legs (not shown). We have observed this type of notch also in fore- and middle-legs of male *M.*  *europaea* bees (not shown).

The oil-collection area in middle-legs extends over the ventral sides of tarsus and tibia and consists exclusively of pluridentate setae. Comparable to setae on the fore-legs, these also form a concave surface with their tips. On the tibia, pluridentate setae are also present in an anterior position (Fig. 3B: “plr,” Fig. 6A) adjacent to the oil-collection area, but in contrary to those in the oil-collection area these have similar lengths, thus not forming a concave structure (Fig. 3B: “plr,” Fig. 6A). The tibia in middle-legs has a roughly triangular cross-section, as described by Vogel (1986) (Fig. 3B).

Comparable to the dorsal sides of the fore-legs the dorsal sides of both tarsomeres and tibia in middle-legs are also covered by plumose setae (Fig. 6B) interspersed by conical and apparently rigid setae. In a posterior position adjacent to the oil-collection, tibiae of the middle-legs are covered by a structure composed of long and stiff setae, which have been described as a pollen brush by Vogel (1986) (Fig. 6B: “pb”).

**Fig. 2.**
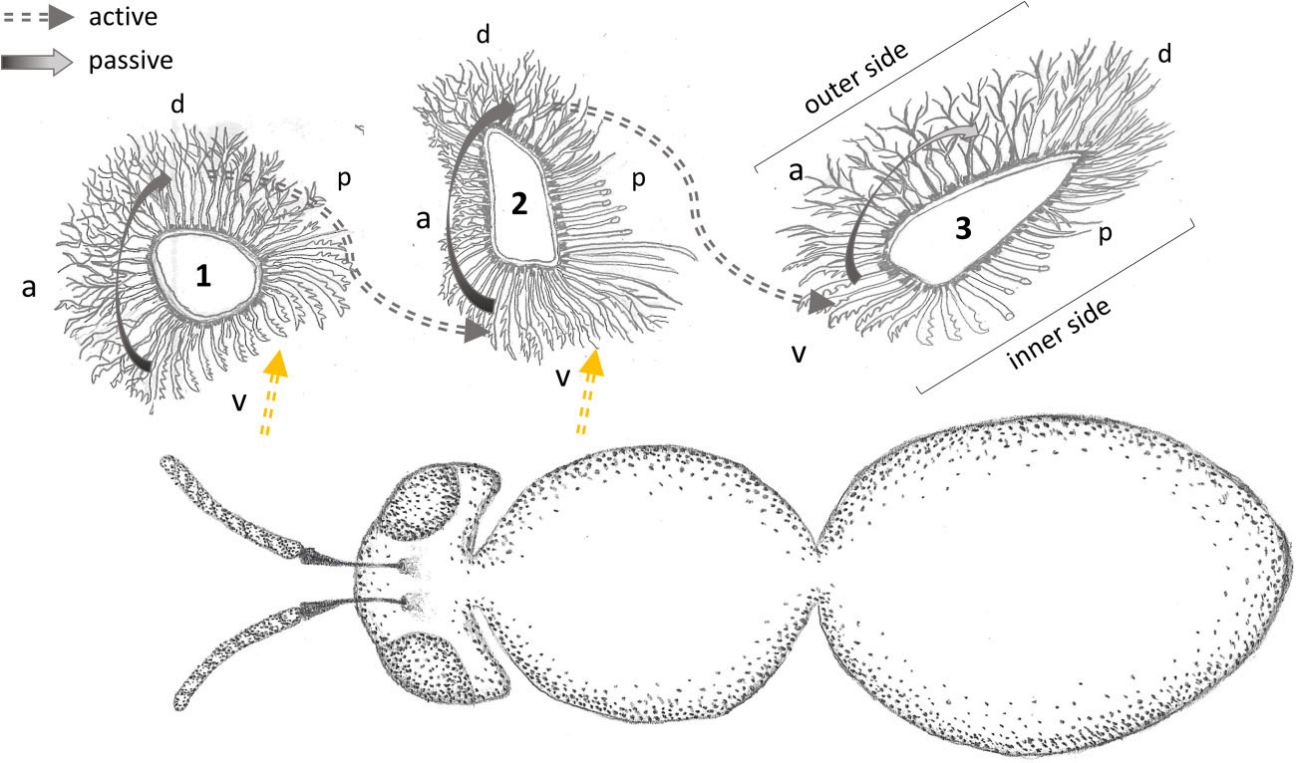
Sketch of cross-sections of the legs of a female *M.*  *europaea* bee and their orientation to the body in resting position. (1) Cross-section of fore-leg tarsal segment. (2) Cross-section of middle-leg tibia. (3) Cross-section of hind-leg tibia. Arrows indicate the locations and positions of uni-directional oil transfer. The dotted arrows on the ventral side represent the direction of the uptake of floral oil from the flower. a, anterior; p, posterior; v, ventral; d, dorsal. Oil transports within a leg occur passively immediately after applying of the oil. Oil transport among legs involves active movements of the legs under natural conditions (or contact with a slight pressure in our experiments, respectively).

Tarsi of hindlegs have an approximately circular cross-section and are covered by pluridentate setae on all sides. These are larger, with longer distal ends, compared to the pluridentate setae of the fore- and middle-legs. Cross-sections in tibia (and femur) in hind-legs are oval and considerably larger than in fore- and middle-legs. In contrast to the fore- and middle-legs, which display an oval (or roughly triangular) cross-section with a longer diameter orientated perpendicularly to the body axis, the large cross-sections of the hind-legs are oval with the longer diameter oriented in parallel to the body axis, resulting in the formation of broad “outer” and “inner” sides (Figs. 2 and 7). The “outer” sides (corresponding to the dorsal sides in fore- and middle-legs) are covered by plumose setae, interspersed with longer and apparently rigid setae (Fig. 4C). The narrow anterior rim of the tibia presents a unique comb-like structure (Fig. 7, tibia comb).

**Fig. 3.**
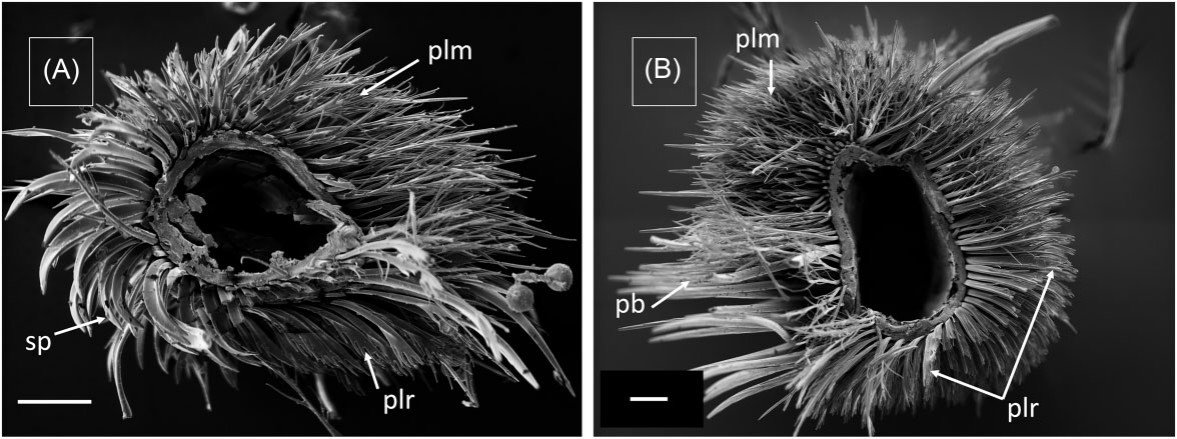
SEM images of leg cross-sections and single setae of a female *Macropis europaea*. (**A**) Fore-leg tarsus with pluridentate setae (plr) and plumose setae (plm) on the dorsal side (top). Spatula shaped setae (sp), found exclusively on fore-legs so far, are visible on the ventral side (bottom). (**B**) Middle-leg tibia with pluridentate setae (plr) on the ventral side (bottom) and anterior side (right), plumose setae (plm) on the dorsal side (top) and the pollen brush (pb) on the posterior side (left). Scale 50 µm.

**Fig. 4.**
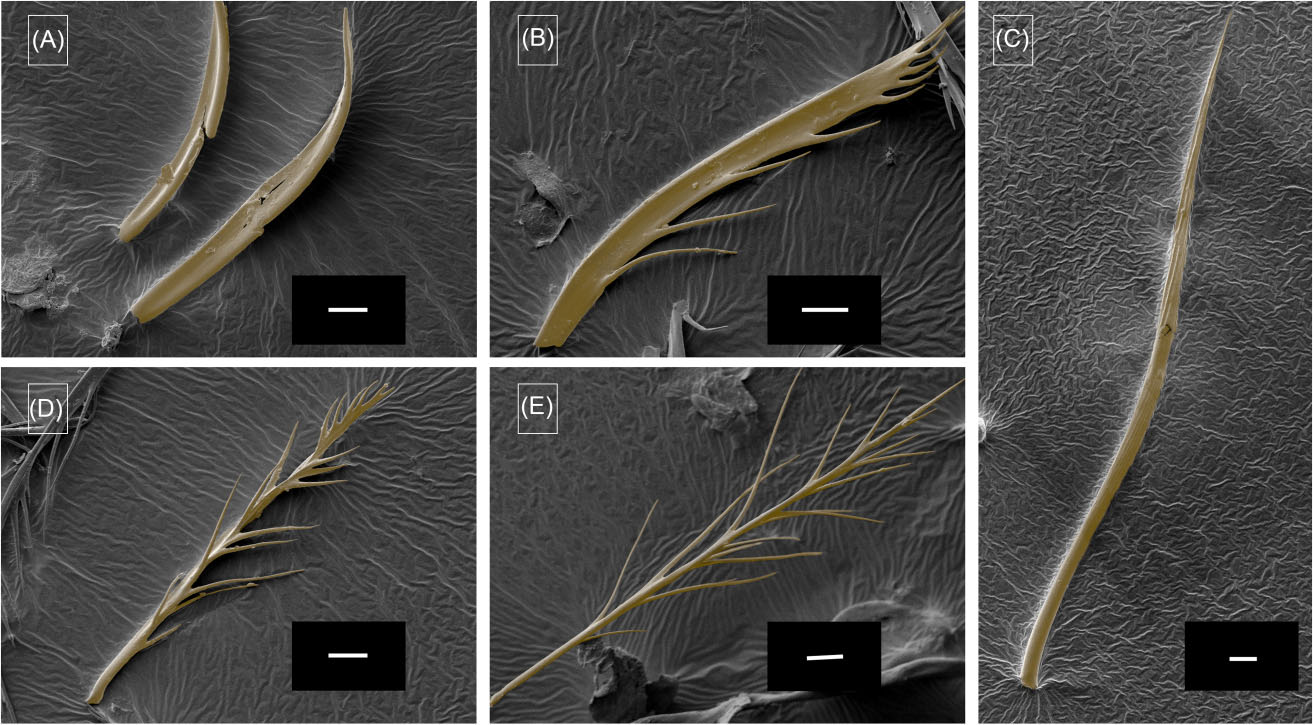
SEM images of different setae types. (**A**) Spatula seta (sp). (**B**) Lamellar pluridentate seta (plr). (**C**) Rigid conical setae. (**D**) Intermediate type seta. (**E**) Plumose seta (plm). Scale 20 µm.

**Fig. 5.**
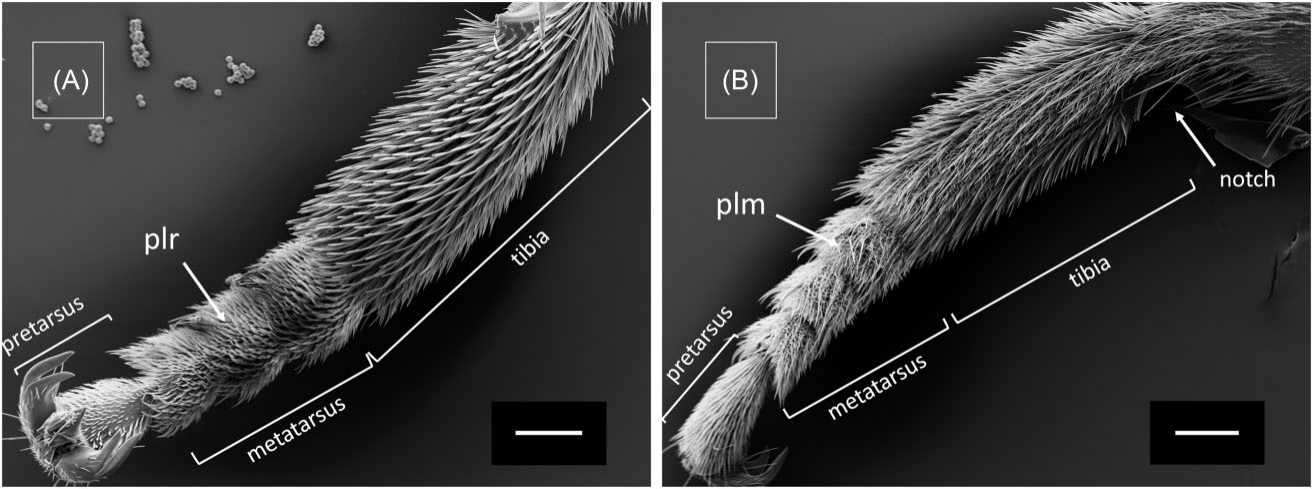
SEM images of a *M.*  *europaea* female's fore-leg. (**A**) Ventral side with pluridentate setae (plr) on the tarsus. Scale 200 µm. (**B**) Dorsal side with plumose setae (plm) covering tarsus and tibia. Scale 200 µm.

**Fig. 6.**
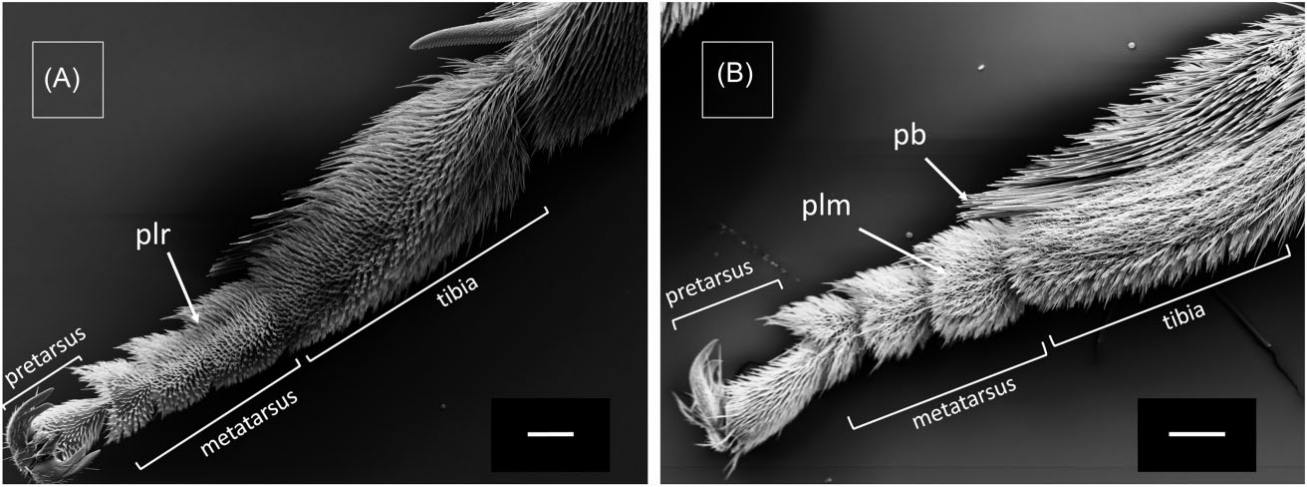
SEM images of a *M.*  *europaea* female's middle-leg. (**A**) Ventral side with pluridentate setae (plr) on the tarsus and tibia. Scale 500 µm. (**B**) Dorsal side with plumose setae (plm) on tarsus and tibia, and pollen bristle (pb) composed of rigid, unbranched single setae. Scale 200 µm.

**Fig. 7.**
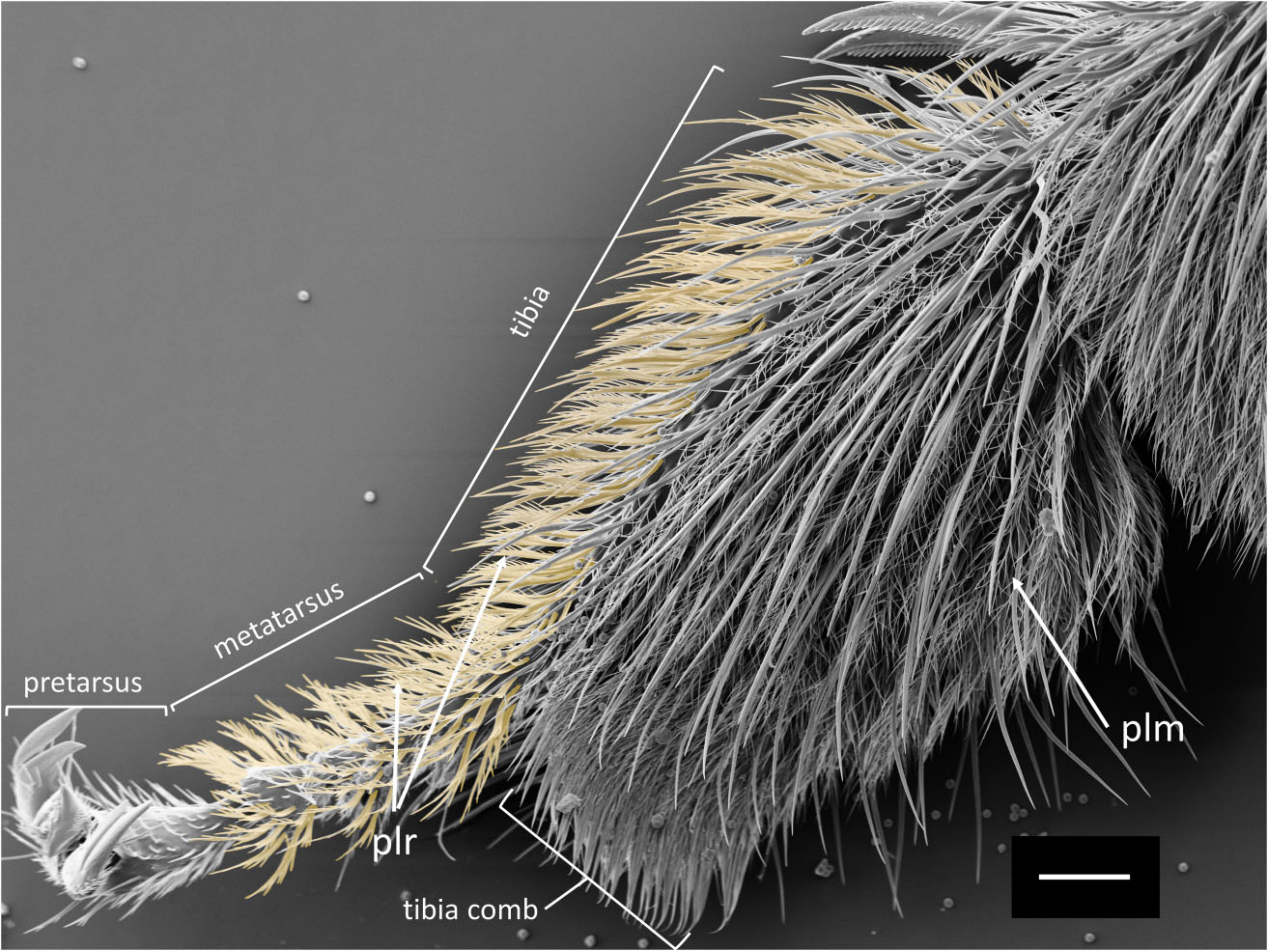
SEM image of a *M.*  *europaea* female's hind-leg with pluridentate setae (plr) on the ventral side (marked in yellow: left side in the image) and plumose setae (plm) covering the dorsal side of the hind-leg. Scale 200 µm.

### Oil transfer processes

#### Unidirectional transport from ventral to dorsal tarsus sides within legs

**Fig. 8.**
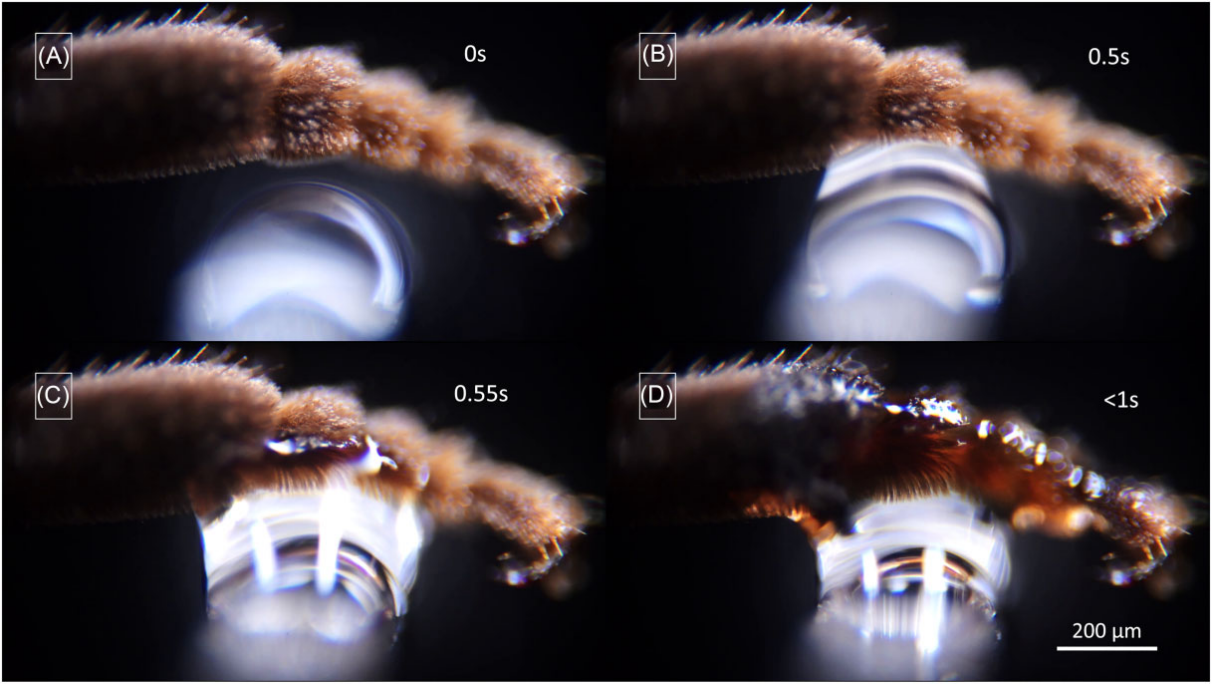
Frames of a Video showing the oil droplet uptake onto a ventral fore-leg side against gravity and rapid oil-distribution onto the dorsal leg side. Video 1a, supplementary section.

**Fig. 9.**
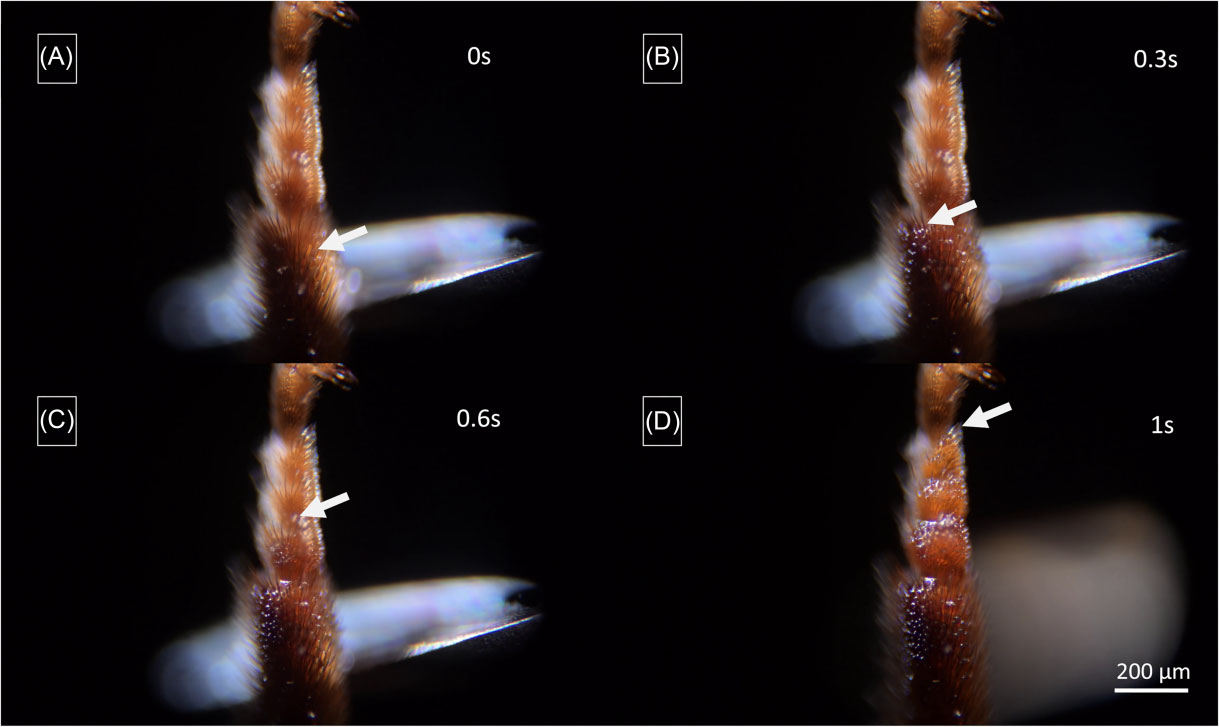
Frames of a Video showing the oil distribution from tibia to tarsus of a fore*-*leg against gravity. Video 1b, supplementary section.

Immediately upon the application of oil to the ventral surface of the fore-leg tarsi, the oil flows uni-directionally to the dorsal side. This flow is independent of the spatial orientation of the bee's leg and also occurs against gravity (Figs. 8, 9, Video 1a, Video 1b, supplementary section).

**Fig. 10.**
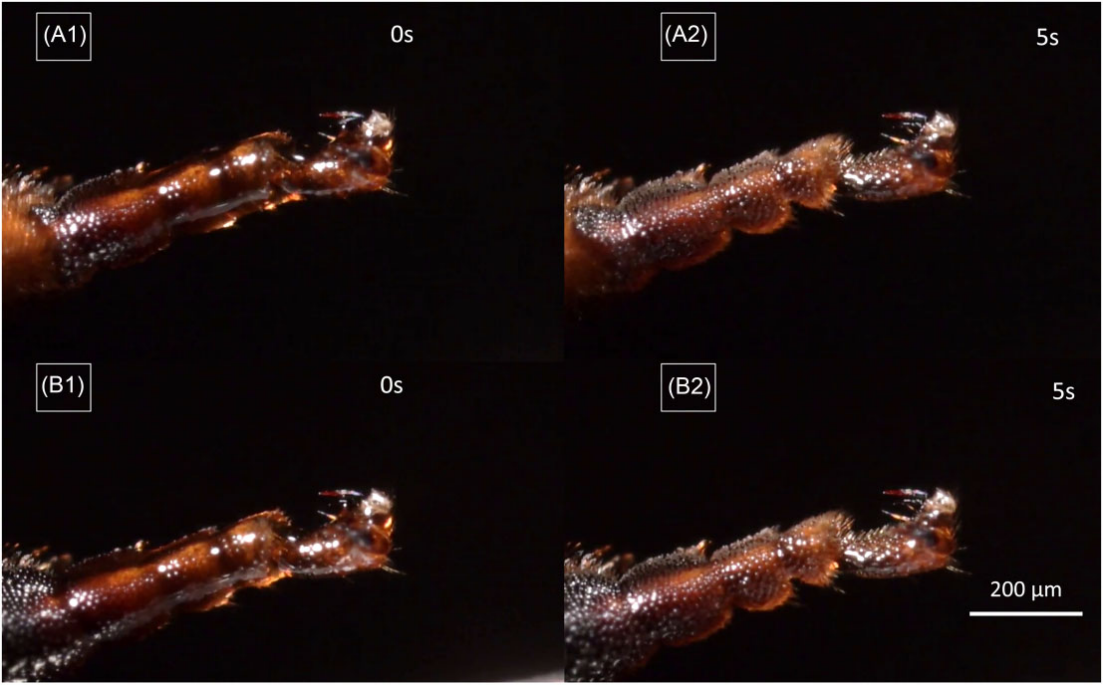
Frames of a Video showing a middle-leg after 2 subsequent oil applications (A1 and B1) and subsequent oil distribution (A2 and B2) away from the ventral oil collector side. Video 2, supplementary section.

Similar processes are observed upon application of oil to the ventral sides of tarsi in middle- and hind-legs, with an immediate movement of the oil to the dorsal sides, respectively. Even after successive loadings with a microsyringe, with up to 2 µL oil each, the ventral surfaces remain with visibly less oil in comparison to other surfaces in front-, middle-, and hind-legs (Fig. 10, Video 2, supplementary section). In hind-legs, oil applied to the narrow ventral side moves immediately to the comb-like structure on the hind-legs tibia (Fig. 13D, white arrow).

#### Transport of remnant oil along setae in fore-legs

After contact of the front- and middle-leg the oil is transferred onto the middle-leg, the small amounts of oil remaining within the mesh of plumose setae on the dorsal sides of tarsi and tibiae of the fore-legs moves along the setae toward their tips.

**Fig. 11.**
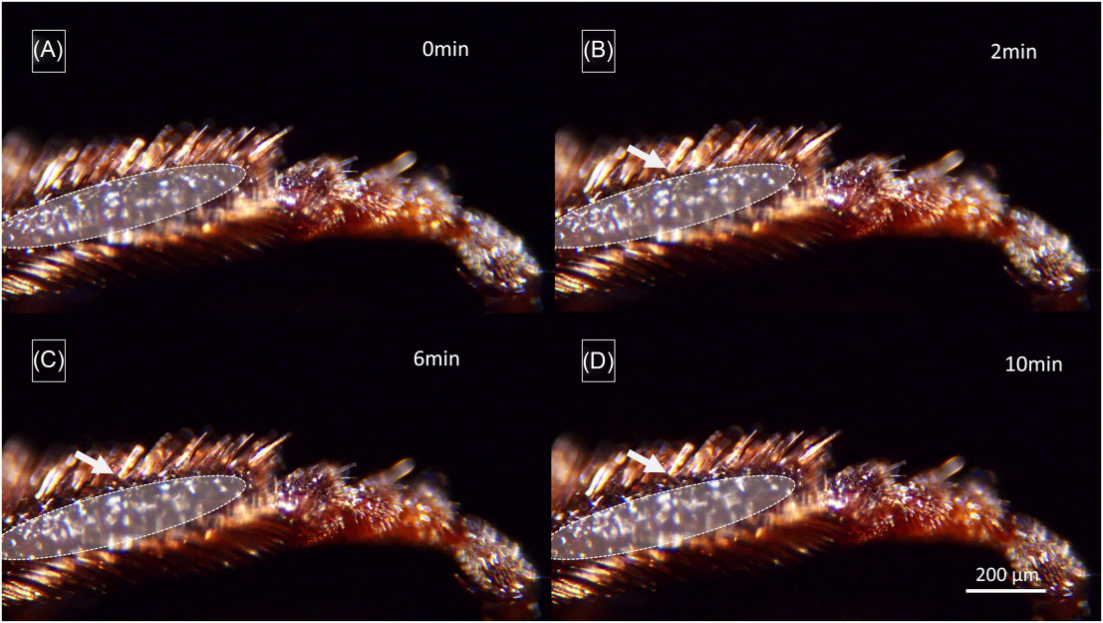
Frames of a Video showing the movements of remnant oil along setae from the bases to their tips. White reference areas correspond to the initial oil area in image A. The white arrow indicates the rise of oil along the setae (total transport duration from base toward tip approximately 7 min). Video 3, supplementary section.

In comparison to the rapid passive transfers of oil from ventral to dorsal leg sides and from leg to leg, this secondary passive transport from the bases toward the tips of single setae is considerably slower. The appearance of rising oil at from the base of the setae is visualized by an increasing dark area (Fig. 11, Video 3, supplementary section).

### Oil transfer from fore-leg to middle-leg

**Fig. 12.**
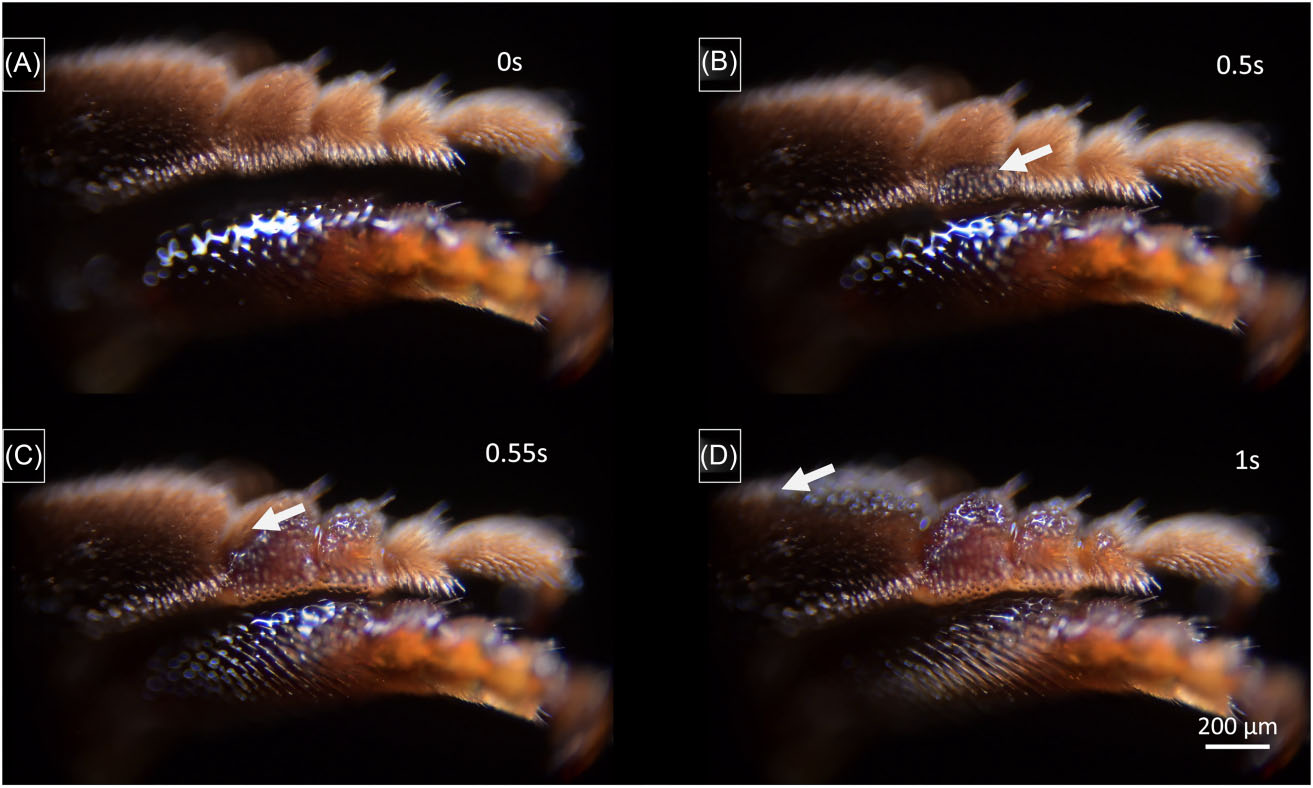
Oil transfer from fore-leg to middle-leg. Following the establishment of contact between an oil-loaded dorsal tarsus and tibia of a fore-leg (below) and an oil-free tarsus and tibia of a middle-leg (above), the oil almost immediately moves against gravity onto the middle-leg. (**A**) Situation before contact. (**B**) Beginning oil transfer immediately after contact establishment. (**C**) and (**D**) Continuing oil distribution onto the tibia's storage structure. White arrows indicate the position of the oil-air interface. (Video 4, supplementary section).

When applying oil to the ventral surface of a fore-leg tarsus, a movement of oil toward the dorsal side can be observed, as described above (Figs. 8  and 9). Upon contact between this oil-loaded dorsal surface of the fore-leg tarsus and tibia and the oil-free ventral tarsus and tibia surfaces of the middle-leg, a rapid transfer of oil occurs at the point of contact. The transfer speed is highest after the initial contact and gradually decreases until the transfer comes to a halt after approximately 5 s (Fig. 12, Video 4, supplementary section). Originating from the point of contact, the oil spreads on all the sides of tarsus and tibia of the middle-leg, excluding the ventral surfaces of the middle-leg (as in the experiments with direct oil-loading of the middle-legs). The oil can be transferred from the tarsomere to the tibia without apparent loss of speed and evenly distributes within the plumose storage hairs on the dorsal side (Fig. 12). Following the first touch between legs, these segments become filled with oil. After a second loading, the storage hairs can still capture almost an entire second load of approximately $0.2\mu {\mathrm{L}}$ oil from the fore-leg, leaving only very little oil behind. After the third loading and the third transfer contact, the gap between the tibia and femur is filled with fluid, forming a capillary bridge, and the leg hairs of the femur also become loaded with oil (not shown).

#### Oil transfer from middle-leg to hind-leg

**Fig. 13.**
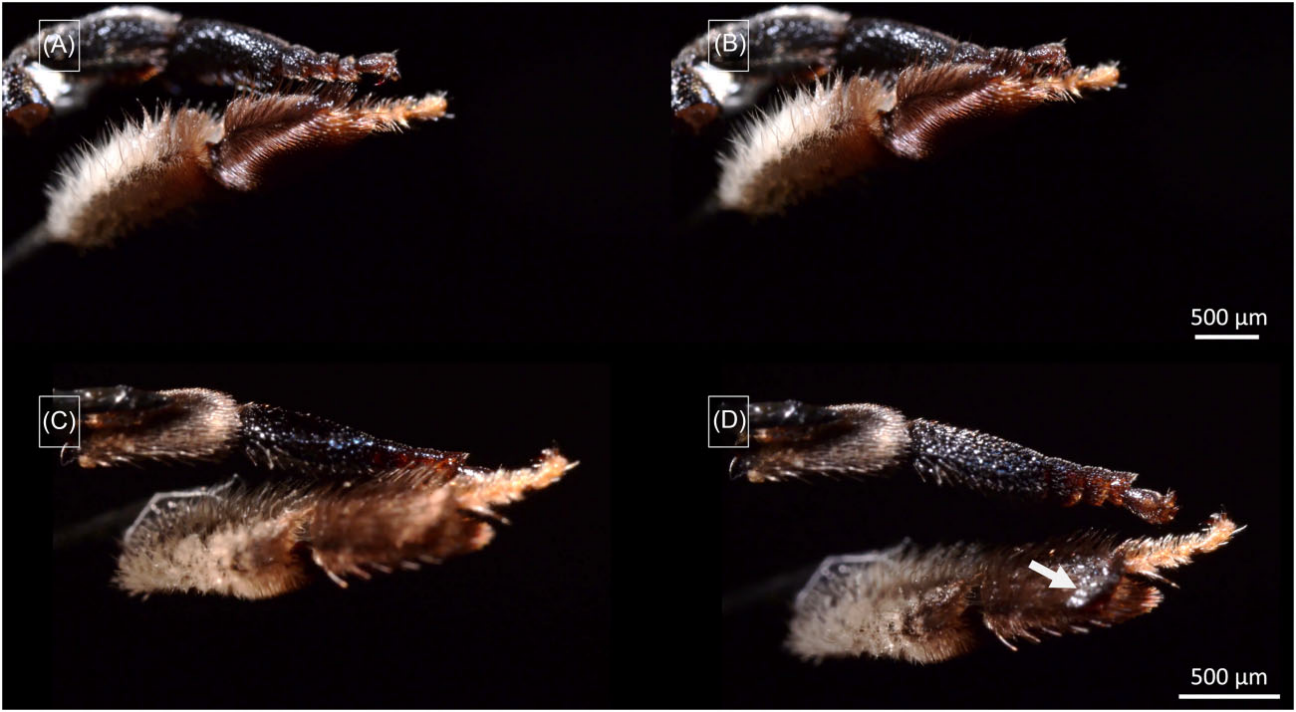
(**A**) and (**B**) Oil transfer from the middle-leg (upper leg in each frame) to the hind-leg (bottom leg) with interaction between the ventral middle-leg surface and the dorsal hind-leg surface, as natural contact caused by bee movement suggested by Vogel (1986). This orientation roughly corresponds to a contact establishment of the legs maintaining their resting positions (Video 5a, supplementary section). (**C**) and (**D**) Oil transfer from the middle-leg (above) to the hind-leg (below) with interaction between the posteroventral middle-leg surface and the ventral hind-leg surface (Video 5b, supplementary section), involving a change of leg position different to the one adopted in resting phases (positioning compared to the one found during cleaning procedures (Video 6, supplementary section). White arrows indicate the rapid oil movement toward the tibia-comb of the hind-leg.

When a middle-leg is loaded with oil and put into contact with a hind leg according to the positions presumably taken during oil transfer according to Vogel (1986, 2002), the oil transfer proved to be slow and inefficient in our experiments (Fig. 13, orientation A and B, Video 5a, supplementary section). This is at least partly due to the fact that the oil on the middle-leg is transported almost immediately after application from the ventral to the dorsal side of the middle-leg (as described above). On the contrary, oil transfer occurs precisely and rapidly, in approximately 6 s, when contact is formed between the dorsal middle-leg side and the narrow ventral side between the “outer” and “inner” side of the hind-leg (pointing forward in resting position) (Fig. 13, orientation C and D, Video 5b, supplementary section). However, orientation B implies a positioning of the legs different from those adopted during the resting phases, a positioning that has so far only been observed during cleaning procedures (Video 6, supplementary section).

Figure 2 summarizes the different kinds of oil transport observed in legs of *M.*  *europaea*.

## Discussion

Vogel (2002) reported that oil transfer occurs between the ventral side of tarsi in fore-legs and dorsal sides of tarsi and tibia in middle-legs. Our observations of *M.*  *europaea* bees visiting *L.*  *vulgaris* flowers and experimental findings identify several different collection and transport ways contradicting this assumption (cf. Fig. 2). After collection with the ventral side of tarsi in fore-legs, the oil is uni-directionally and passively transferred to the dorsal sides of tarsi. The transfer to middle-legs happens when the dorsal side of fore-leg tarsi get actively in contact with the ventral side of middle-legs tarsi and tibiae. Our experiments confirm that uni-directional oil-transport takes place in the described contact position. In this scenario, the plumose setae of the dorsal sides release the oil, while the pluridentate setae of the ventral sides of the next leg absorb it. The same applies for the structures present in the middle-legs, resulting in a transport from the ventral parts to the dorsal parts of the tarsi and tibiae, entailing a probable oil transfer from these dorsal parts to the hind-legs. The uni-directional oil transport within the hind-legs from the oil-receiving ventral side toward the comb-like structures on the tibiae brings the oil into its final storage position. A schematic drawing of the entire process is proposed in Fig. 2.

There are 2 possible mechanisms underlying the observed phenomenon of uni-directional oil transport within the single legs. First, different types and densities of setae in different setae-pads could create a capillarity difference that causes the observed transports of the oil from ventral to dorsal leg sides. Second, the oil could be transferred within the setae network due to the specialized and conical shapes, similar to the passive transport along conical wires or cactus spines (Quéré 2004; Ju 2012) and cross-sectional geometries of the individual setae along the chitin skeleton of the corresponding leg. A similar process has been reported for the passive water collection by the horn scales of the Australian horned lizard [described as a liquid diode by Comanns et al. (2015)]. This may correlate with the different geometric properties observed between different setae types and also within different parts of single setae. A combination of both mechanisms is possible. The quantitative analyses of these mechanisms and of their potential combination will be part of further studies. These will include experiments on isolated single setae with an atomic force microscope, an analysis of the setae material composition, the establishment of an experimental setup allowing for estimations of the contact angles formed by the oil droplets and of the setae array porosity.

Vogel (1986) suggested a capillary gradient driving the transfer of oil from leg to leg during backloading processes. Although fore-, middle-, and hind-legs differ in their morphology, they share a common feature in distribution of setae types, with pluridentate setae on ventral sides and plumose setae on dorsal sides of tarsi and/or tibiae. Although a capillary gradient could play a role in the observed transport from ventral to dorsal sides within a leg, occurring uni-directionally from pluridentate to plumose setae, it seems difficult to imagine a capillary gradient also driving oil-transport in the opposite direction, from the plumose setae of one leg to the pluridentate setae of the next leg. We therefore hypothesize that the functional principles involved in the oil-transport from leg to leg are different from the uni-directional transports within one leg. The exact nature and role of the highly complex movements performed by the bees during the backloading processes from fore-leg to middle-leg and finally to the storage structures of the hind-leg remain to be elucidated.

The structure-function-relations of the notches and serrated comb-likes structures present between the tibia and femur in varying forms on the fore-, middle-, and hind-legs of female *M.*  *europaea* reported by Vogel (1986, 2002) also remains unclear. In our experiments, they do not seem to play a dominant role in the processes of oil-transport between different legs. This is further supported by the presence of these notches in legs of male *M.*  *europaea* bees, who do not collect and backload floral oil. It is conceivable that these notches rather play a role in cleaning processes, to get rid of accidentally attached pollen or other particles. Another question that remains to be elucidated is how the female *M.*  *europaea* bees get rid of the pollen-oil mixture stored on their hind-legs in the brood cells, and how the (probably viscoelastic) properties of this mixture influence the process, as described by Matherne et al. (2021) for pollen-oil pellets and their removement in honey bees.

Finally, the complex interplay of passive, uni-directional oil-transport from ventral to dorsal leg sides with onward transport to the next leg (probably involving an active energy input from the bee) results in both an increasing volume of stored oil on the hind leg and a simultaneous cleaning of the collection structures of the fore-leg, thus available for the next oil collection. Our study is the first to show the involvement of passive unidirectional transport from one leg side to the other, and active transport from leg to leg. These findings, on closer inspection, differ fundamentally from those described by Vogel (1986, 2002), and will help to elucidate which setae types enable these different processes. However, our results were obtained under simplified conditions in the laboratory and a one-to-one transferability into the complex situation in a living bee can't be assumed. Thus, for instance, living insects cover their exoskeleton with lipids and the viscosity of the native floral oil might differ from that of the sunflower oil used in our experiments. Additionally, the leg movements executed by the bee are highly complex, resulting in different relative angles and contact pressures between touching legs, and probably happen at different speeds. All these aspects presumably influence the oil behavior on the surface.

Nevertheless, the experiments under simplified conditions in the laboratory are a prerequisite not only for a better understanding of the processes in nature but also for any potential transfer to bioinspired technical material systems. The oil transport processes and the functional morphology of the setae in *M.*  *europaea* have a high potential as concept generators for future biomimetic developments. This holds in particular for the passive uni-directional oil transports, which render the involved structures clean and functional again as oil acceptors, as described in this study. However, further detailed analyses of the physico-chemical properties including measurements in single setae are needed as a prerequisite for such developments and are studied in ongoing experiments.

## Supplementary Material

obaf025_Supplemental_File

## Data Availability

Analyses reported in this article can be reproduced using the videos provided in the supplementary materials.

## References

[bib2] Buchmann SL. 1987. The ecology of oil flowers and their bees. Annu Rev Ecol Syst 18:343–69. 10.1146/annurev.es.18.110187.002015

[bib4] Celary W. 2004. A comparative study on the biology of *Macropis* *fulvipes* (Fabricus, 1804) and *Macropis* *europaea* Warncke, 1973 (Hymenoptera: apoidea: melittidae). Folia Biol 52:81–5. 15521653

[bib5] Comanns P, Buchberger G, Buchsbaum A, Baumgartner R, Kogler A, Bauer S, Baumgartner W. 2015. Directional, passive liquid transport: the Texas horned lizard as a model for a biomimetic ‘liquid diode‘. J R Soc Interface 12:20150415. 10.1098/rsif.2015.0415 26202685 PMC4535411

[bib6] Jesus BMV, Garofalo CA. 2000. Nesting behaviour of *Centris* (Heterocentris) *analis* (Fabricus) in southeastern Brazil (Hymenoptera: apidae: centridini). Apidologie 31(4):503–15. 10.1051/apido:2000142

[bib7] Ju J, Bai H, Zheng Y, Zhao T, Fang R, Jiang L. 2012. A multi-structural and multi-functional integrated fog collection system in cactus. Nat Commun 3(1):1247. 10.1038/ncomms2253 PMC3535335 23212376

[bib8] Kuhlmann M. 2014. Nest architecture and use of floral oil in the oilcollecting South African solitary bee *Rediviva* *intermixta* (cockerell) (Hymenoptera: apoidea: melittidae). Jour Nat Hist 48 (43-44):2633–44. 10.1080/00222933.2014.909069

[bib9] Kuhlmann M, Hollens H. 2015. Morphology of oil-collecting pilosity of female *Redivia* bees (Hynmenoptera: apoidea: mellitidae) reflects host plant use. Jour Nat Hist 49:561–73. 10.1080/00222933.2014.939732

[bib10] Lorenceau E, Quéré D. 2004. Drops on a conical wire. J Fluid Mech 510:29–45. 10.1017/S0022112004009152

[bib11] Martins AC, Aguiar AJC, Alves-Dos-Santos I. 2013. Interaction between oil-collecting bees and seven species of Plantaginaceae. Flora—Morphol, Distrib, Funct Ecol Plants 208:401–11. 10.1016/j.flora.2013.07.001

[bib12] Matherne M, Dowell-Equivel C, Howington O, Lenaghan O, Steinbach G, Yunker PJ, Hu L. 2021. Biomechanics of pollen pellet removal by the honey bee. J R Soc Interface. 18(181):20210549. 10.1098/rsif.2021.0549. 34428943 PMC8385348

[bib13] Neff JL, Simpson BB. 1981. Oil-collecting structures in the Anthophoridae (Hymenoptera): morphology, function and use in systematics. J Kan Ent Soc 54(1):301–22.

[bib14a] Renner SS., Schaefer H. (2010). The evolution and loss of oil-offering flowers: new insights from dated phylogenies for plants and bees. Philos Trans R Soc B, Biol Sci. 365:423–35. 10.1098/rstb.2009.0229 PMC2838259 20047869

[bib14] Renner SS. 2021. Evolution: how flowers switch from nectar to oil as a pollinator reward. Curr Biol 31:R18–20. 10.1016/j.cub.2020.10.057 33434479

[bib15] Schäffler I, Dötterl S. 2010. A day in the life of an oil bee: phenology, nesting and foraging behavior. Apidologie 42:409–24. https://doi.org/1007/s13592-011-0010-3

[bib16] Simpson BB, Neff JL, Dieringer G. 1990. The production of floral oils by *Monttea* (Scrophulariaceae) and the function of tarsal pads in *Centris* bees. Plant Syst Evol 173:209–22. 10.1007/BF00940864

[bib17] Vinson B, Frankie GW, Williams HJ. 1996. Chemical ecology of bees of the genus *Centris* (Hymenoptera: apidae). Fla Entomol 79(2):109. 10.2307/3495809

[bib18] Vogel S. 1974. In: Rauh, W editors. Ölblumen und Ölsammelnde Bienen. Trop. Subt. Pflanz. 7. Akademie der Wissenschaften und der Literatur Mainz. Stuttgart: Franz Steiner Verlag Wiesbaden GmbH. p. 267. 10.1002/fedr.19770880110

[bib19] Vogel S. 1986. In: Rauh, W, editors. Ölblumen und Ölsammelnde Bienen II. Trop. Subt. Pflanz. 54. Akademie der Wissenschaften und der Literatur Mainz. Stuttgart: Franz Steiner Verlag Wiesbaden GmbH. p.186. 10.1002/fedr.19770880110

[bib20] Vogel S. 2002. Yellow loosestrife and hair-legged mining bees (*Lysimachia* and *Macropis*). Ölblumen und ölsammelnde Bienen, *IWF**Göttingen*. Video: https://av.tib.eu/media/18827. 10.3203/IWF/W-7049eng [accessed 2024 June 20].

